# Immediate Impact of an 8-Week Virtual Reality Educational Program on Burnout and Work Engagement Among Health Care Professionals: Pre-Post Pilot Study

**DOI:** 10.2196/55678

**Published:** 2024-04-25

**Authors:** Jose Ferrer Costa, Nuria Moran, Carlos Garcia Marti, Leomar Javier Colmenares Hernandez, Florin Radu Ciorba Ciorba, Maria Jose Ciudad

**Affiliations:** 1 Innovation and Projects Department Badalona Serveis Assistencials Badalona Spain; 2 Primary Care Service Badalona Serveis Assistencials Badalona Spain; 3 Centre Sociosanitari El Carme Badalona Serveis Assistencials Badalona Spain; 4 Department of Clinical Psychology and Psychobiology Faculty of Psychology Universitat de Barcelona Barcelona Spain

**Keywords:** virtual reality, burnout, mindfulness, health care professionals, mental health, health promotion, educational intervention

## Abstract

**Background:**

Health care professionals globally face increasing levels of burnout characterized by emotional exhaustion, depersonalization, and a reduced sense of accomplishment, and it has been notably exacerbated during the COVID-19 pandemic. This condition not only impacts the well-being of health care workers but also affects patient care and contributes to significant economic burden. Traditional approaches to mitigating burnout have included various psychosocial interventions, with mindfulness being recognized for its effectiveness in enhancing mental health and stress management. The emergence of virtual reality (VR) technology offers a novel immersive platform for delivering mindfulness and emotional management training.

**Objective:**

This study aimed to evaluate the immediate impact of an 8-week VR educational program on burnout and work engagement among health care professionals.

**Methods:**

This nonrandomized pre-post intervention study enrolled 90 health care professionals, including nurses, physicians, and allied health staff, from 3 different centers. Of these 90 professionals, 83 (92%) completed the program. The intervention consisted of 8 weekly VR sessions of 10-13 minutes each, using Meta Quest 2 headsets. The sessions focused on mindfulness and emotional management. The Maslach Burnout Inventory (MBI) and Utrecht Work Engagement Scale (UWES) were used for assessments. Data analysis involved inferential statistical techniques for evaluating the impact on the scales, including paired *t* tests for normally distributed variables and Wilcoxon signed rank tests for nonnormally distributed variables. The significance of changes was indicated by *P* values <.05, with effect sizes measured using Cohen *d* for *t* tests and Cohen *r* for Wilcoxon tests for quantifying the magnitude of the intervention’s effect.

**Results:**

The statistical analysis revealed significant improvements in the MBI and UWES indices after the intervention (*P*<.05). Specifically, the MBI showed reductions in emotional exhaustion (*t*_82_=5.58; *P*<.001; Cohen *d*=0.61) and depersonalization (*t*_82_=4.67; *P*<.001; Cohen *d*=0.51), and an increase in personal accomplishment (*t*_82_=−3.62; *P*<.001; Cohen *d*=0.4). The UWES revealed enhancements in vigor (*t*_82_=−3.77; *P*<.001; Cohen *d*=0.41), dedication (*Z*=−3.63; *P*<.001; Cohen *r*=0.41), and absorption (*Z*=−3.52; *P*<.001; Cohen *r*=0.4).

**Conclusions:**

The study provides initial data supporting the effectiveness of VR-based educational programs for reducing burnout and enhancing work engagement among health care professionals. While limitations, such as the absence of a control group, are acknowledged, the significant improvements in burnout and engagement indices coupled with high participant adherence and minimal VR discomfort underline the potential of VR interventions in health care settings. These encouraging findings pave the way for more comprehensive studies, including randomized controlled trials, to further validate and expand upon these results.

## Introduction

### Background

Burnout among health care workers is a growing concern that affects professionals worldwide. Burnout is characterized by emotional exhaustion, depersonalization, and a diminished sense of personal accomplishment [1], and it extends beyond transient workplace stress, potentially undermines patient care quality, increases the rate of errors, creates a negative work climate, and contributes to health care costs [1-3]. The prevalence of burnout varies considerably, with a scoping review during the COVID-19 pandemic reporting rates of 4.3% to 90.4% among health care workers, reflecting the significant mental burden and high levels of stress and burnout experienced by frontline health care workers during this period [4]. This review highlighted multiple factors associated with increases or decreases in burnout, including demographic characteristics, psychological conditions, social factors, work organization, and direct COVID-19–related impacts, providing valuable insights for policy makers and health care managers [4].

The COVID-19 pandemic has notably worsened this panorama, causing unprecedented spikes in burnout rates. Data from Spain [5] revealed an increase from 10% before the pandemic to a staggering 50% during the pandemic, with a marked rise in emotional exhaustion and depersonalization, and a decline in personal accomplishment among primary care physicians.

Economically, burnout contributes to substantial costs in the health care system, with estimates in the United States indicating a US $979 million annual burden related to turnover and reduced clinical hours among primary care physicians alone [6,7]. To deal with this issue, the World Health Organization has underscored the importance of psychosocial interventions, including mindfulness and cognitive behavioral strategies, to enhance mental health and stress management in the workplace [8].

Contemporary research supports mindfulness as an effective intervention against burnout [9,10]. This practice, originally encapsulated by Kabat-Zinn’s [11] conceptualization as an intentional nonjudgmental focus on the present experience, is gaining recognition as a cornerstone in the arsenal against burnout among medical professionals [12]. The implementation of mindfulness is corroborated by evidence indicating its significant contribution to bolstering emotional resilience, fostering effective communication, and reinforcing collaborative dynamics in the often high-pressure clinical environment [13-15].

### Integration of Mindfulness Practices in Health Care Through Virtual Reality Technology

With the integration of technology into health care, virtual reality (VR) offers a novel platform for mindfulness training. By simulating controlled environments, VR can deepen mindfulness practices, potentially surpassing traditional methods in improving mood, sleep quality, and cognitive focus [16]. The immersive experiences of VR are known to increase knowledge retention and engagement, making educational interventions more effective [17-22].

The application of VR to mindfulness training presents a unique opportunity for health care professionals to cultivate skills for managing stressors inherent to their profession. Empirical evidence suggests that VR-based mindfulness can yield significant benefits for emotional regulation and stress reduction, thus improving the well-being of health care providers [23-26].

### Study Objective and Hypothesis

The objective of this pilot study was to evaluate the immediate impact of an 8-week VR educational program on burnout and work engagement among health care professionals. The study measured changes in the levels of emotional exhaustion, depersonalization, and personal accomplishment as characterized by the Maslach Burnout Inventory (MBI), along with the dimensions of vigor, dedication, and absorption as depicted by the Utrecht Work Engagement Scale (UWES).

The study was guided by 2 hypotheses. The null hypothesis (H0) proposed that the VR educational program would not result in significant changes in burnout or work engagement levels, and any variations could be attributed to random fluctuation rather than the intervention’s effect. Conversely, the alternative hypothesis (H1) suggested that the VR program would lead to significant improvements in these measures, reflecting a direct positive impact of the intervention on the professional well-being of participants.

While burnout is recognized as a multifaceted syndrome influenced by a variety of factors, the project posited that a targeted VR program focusing on mindfulness and emotional management might enhance resilience among health care professionals. This resilience, in turn, could modify their perception and coping mechanisms, equipping them with effective strategies to counteract work-related stress. The insights gained from this study are expected to contribute to the development of innovative and more personalized interventions that are tailored to the needs of health care workers [25,26].

## Methods

### Study Design

In this prospective interventional pilot study, a nonrandomized pre-post intervention design was used to explore the preliminary effects of a mindfulness-based VR educational program in health care professionals.

### Participants and Setting

This pilot study enrolled 90 health care professionals, representing a broad range of specialties in the health care sector. The demographic profile of the 83 participants who completed the program revealed an average age of 46.39 years and a mean professional tenure of 17.57 years. The cohort was predominantly female, with only 7 male participants, reflecting the sex distribution that is common in these centers. The detailed breakdown of professional roles within the cohort is presented in the Results section.

### Selection and Enrollment

#### Recruitment

Recruitment for the study was conducted via an open invitation across 3 health care centers, and the study targeted a diverse group of professionals, including nurses, physicians, administrative staff, nursing assistants, occupational therapists, social workers, psychologists, pediatricians, dentists, and ward assistants.

The first, second, and third centers have been identified in this study as CAP-A, CAP-B, and CSSC, respectively. Informative sessions were held in each center during the recruitment of each group owing to different time periods. During these sessions, all professionals were able to ask questions about VR and the program, and later decide if they were interested in participating. If interested, they were required to send an email to our team to be evaluated for inclusion.

The absence of a control group in this study was a strategic choice, which was influenced by the exploratory nature of the pilot study and resource limitations. This choice and its implications are further discussed in the Limitations subsection. The study’s pre-post design without a control group necessitated a sample size that was feasible within the available resources and anticipated participant availability.

#### Inclusion and Withdrawal Criteria

Health care professionals who were currently employed in the study centers, older than 18 years, and able to commit to the program’s full duration were considered for inclusion. Informed consent was required, along with a commitment to complete all questionnaires. Participants were excluded if they had participated in a similar program or received specific training in burnout prevention within the last 12 months, had a long-term absence from work or any situation that prevented regular attendance during the study, or had vertigo, epilepsy, or significant visual/auditory disabilities that precluded the use of VR glasses. Withdrawal from the study was considered when participants had significant intolerance to VR equipment or were absent for more than two sessions. To facilitate participation and minimize dropout, participants were allowed a 2-week grace period after the program to cover any missed sessions.

#### Sequential Enrollment, Center Allocation, and Attrition

Enrollment and participation were conducted in sequential phases to accommodate the limited number of VR headsets, with only 2 headsets available for the entire study. The initial enrollment occurred at the first center (CAP-A) in February 2022, with 29 participants included and then divided randomly into 3 groups for logistic reasons. The groups completed the program at different times throughout the year, with the final group concluding in December 2022.

Building on the lessons learned from the initial phase regarding resource utilization and scheduling, the process was refined for subsequent enrollments. In 2023, a more streamlined approach was adopted at the next 2 centers, where all participants were enrolled in a single group per center to simplify logistics. Recruitment for the study was conducted simultaneously at both centers in January and February 2023. At CSSC, 41 participants were included, with 36 participants successfully completing the program between February and April 2023. At CAP-B, 20 participants were initially enrolled, with 14 participants completing the program in April and a small additional group of 4 participants completing the program in October 2023.

### Instruments

The intervention was delivered through Meta Quest 2 VR headsets (Meta Platforms, Inc), which were chosen for their cable-free stand-alone functionality and high-quality audiovisual output. The headsets feature hand tracking capabilities that were used in the program to enhance immersion by visualizing users’ hands in VR and allowing simple selection of sessions via the menu. The program was developed between April 2021 and January 2022 following a methodical approach based on bibliographic review and guided by the expertise of author NM. It integrated mindfulness and emotional management techniques, which are well-established methods for reducing burnout and enhancing work engagement, and are particularly beneficial in health care settings [9-26]. The VR sessions were developed by author JFC. VR technology was integrated with the author’s clinical expertise in mindfulness to create a dynamic learning environment. Unity (Unity Technologies) and Blender (Blender Foundation) were used to construct 3D animations that visually represent and illustrate mindfulness and emotion management techniques. These animations serve as immersive educational tools within VR environments, enhancing the learning experience beyond traditional relaxation sessions. Moran Bueno, who specializes in mindfulness at the University of Barcelona, ensured that the content was scientifically accurate and pedagogically effective. The development process involved iterative feedback from health care professionals, aiming to refine the program’s educational impact and comfort. The VR environments were specifically crafted for passive educational engagement, allowing participants to immerse in the program without the need for active interaction or physical movement and thus enhancing the learning experience in a safe and user-friendly manner. The VR program allowed for a more engaging and potentially more impactful learning experience by placing participants in various virtual environments that can enhance the absorption of the techniques taught.

**Figure 1:**
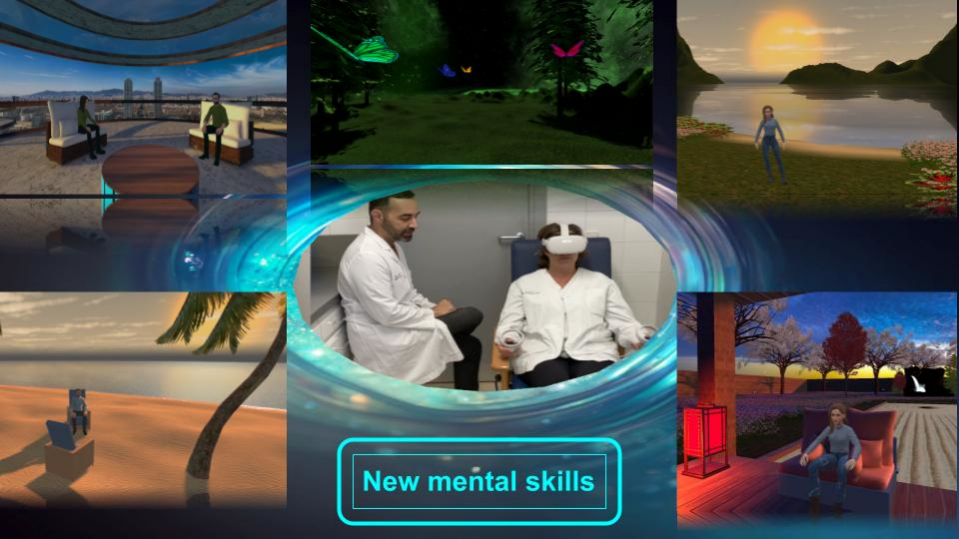
Some of the virtual reality environments of the educational program. Displayed images from the top middle in clockwise direction are butterflies in a garden, a serene bay, a zen garden, a tropical beach, and a terrace with panoramic views of Barcelona. At the center is an image of a supervised session in progress.

The VR sessions immersed participants in various carefully designed settings, such as a serene beach, calm lake, and peaceful Zen garden. Ambient sounds corresponding to each environment, like the soothing lapping of waves, were incorporated to deepen the sensory experience. This auditory enhancement, in tandem with visual elements, augmented the overall educational and immersive quality for the users. The integration of these sensory aspects aimed to create an optimal learning environment for engaging with the mindfulness and emotional management content (Figure 1).

### VR Considerations

Although VR is a technological tool with minimal risk, the use of VR headsets, especially for extended periods, can occasionally lead to side effects, such as cybersickness, eye strain, emotional reactions, and physical danger, in some individuals [20,27-30]. These potential effects were carefully considered in the design of the intervention to minimize any adverse outcomes. The potential side effects are as follows [27,30]:

- Cybersickness: Symptoms include nausea, balance issues, disorientation, headache, eye strain, and general fatigue, which tend to dissipate shortly after the discontinuation of VR use.
- Eye strain: Eye discomfort may occur with extended VR use, although such instances are uncommon given the brevity of typical exposure times in a controlled research setting.
- Intense emotional reactions: The compelling realism of VR and the emotion-related content can provoke emotional responses, requiring monitoring.
- Physical danger: While rare in a controlled environment, there is a potential risk of physical injury due to loss of spatial awareness while immersed in VR. This includes the possibility of tripping, bumping into objects, or other accidents, particularly if the physical space is not adequately prepared for VR activities.

### Mitigation Measures

The project development incorporated strategic measures to address human factors and ergonomic considerations, aiming to minimize VR-induced side effects. These measures, which aligned with current guidelines and best practices, were meticulously designed to ensure the safety and efficacy of the VR intervention [27-30]. The design aspects are as follows:

- Session duration: VR sessions are capped at 10-13 minutes, reducing the risk of cybersickness and visual fatigue [27].
- Static interaction: Design of the VR experience for seated participants (minimal requirement for movement) reduces the risks of nausea and physical activity in VR [28,29].
- Hand tracking: Incorporation of hand tracking technology allows participants to maintain a sense of agency and immersion in the VR environment without necessitating complex interactions [28,29].
- Optimization of the VR content: VR content is carefully designed to avoid overly intense stimuli and to support user comfort. Adjustments to lighting, motion, and frame rates are considered to prevent disorientation and discomfort [27,30].
- Stress relief features: Elements, such as biophilic designs, soft colors, and calming music within the VR environment, are incorporated to create a restorative virtual space for users [27].
- Monitoring and support: Continuous observation during VR exposure and immediate follow-up after VR exposure permits the research team to identify and mitigate any adverse effects. Participants are advised on headset adjustment and encouraged to take breaks as needed [27,29].
- User training: Prior to the initiation of the VR educational program, participants need to undergo a training session focused on VR utilization. This session includes instructions on the proper adjustment of the VR headset, acclimatization to immersive experiences, navigation of the VR interface, and response to potential side effects. The aim is to foster participant independence in managing the VR system and to ensure comfort during use [29].

This approach not only aids in minimizing potential motion-induced discomfort but also ensures a consistent and comfortable experience for all participants. During the course of the research, no VR-related side effects were observed. The only concern reported by a small subset of participants (n=7) was discomfort attributed to the weight of the headset. In most instances, this was alleviated, at least partially, by readjusting the headset. Regular check-ins were conducted after the sessions to monitor any adverse effects, and guidance was provided to participants on taking breaks or adjusting the headset position as needed, which further contributed to the positive reception and comfort of the intervention.

### Procedure

The program lasted for 8 weeks. A calendar was created, and each participant was assigned a specific day and time every week for their 13-minute VR session. Each session was designed with a clear structure, consisting of 3 phases: preparation, VR experience, and cleanup. In order to ensure a secure and efficient implementation of the program, the sessions were conducted under the supervision of one of the researchers. During the preparation phase, participants were welcomed and assisted with the VR headset adjustment. The role of the researcher included monitoring the implementation for consistency, managing any technical issues, and ensuring that the VR equipment was used safely and effectively.

A key advantage of this VR program is that it constitutes a fully self-contained system. The VR content was directly uploaded to the headsets, thereby eliminating dependency on internet connectivity, an essential feature for accommodating health care professionals operating in environments where internet access may be limited or nonexistent.

### Content Overview

Over the sessions, participants engaged with content from the following primary areas:

- Mindfulness training: In these sessions, exercises are focused on breath and body scanning, aiming to ground participants in the present moment. By maintaining an attitude of acceptance and nonjudgment, these sessions aim to teach how to foster mental tranquility.
- Emotional regulation: In these sessions, participants are introduced to techniques designed to address and manage intense emotions. Instead of suppressing or avoiding discomforting feelings, participants are encouraged to approach them with openness and curiosity, potentially deriving insights and understanding from their observations.
- Self-compassion: This area underscores the importance of treating oneself with kindness, especially during challenging times. Recognizing and minimizing the influence of one’s inner critic is crucial. Participants are taught to treat themselves with the same warmth and understanding they would extend to a close friend or loved one, promoting a balanced self-view.

### Variables

The study evaluated the intervention’s impact using demographic and psychometric assessments. Demographic data collected included age, sex, professional category, and tenure, to understand sample characteristics and control for confounding factors. Psychometric evaluation was performed with 2 established tools: MBI and UWES. These 2 scales were administered on paper before the commencement of the first session and after the conclusion of the last session. Both scales score their 3 subdimensions on a Likert scale from 0 (never) to 6 (daily).

The MBI is an established instrument for assessing burnout in health care settings, and it uses 3 subscales, namely, emotional exhaustion, depersonalization, and personal accomplishment, to capture burnout dimensions. Its high reliability has been confirmed with Cronbach α coefficients ranging from 0.71 to 0.90 in the Spanish health care context, validating its use for this study [31-36].

The subscales are interpreted as follows [36]:

- Emotional exhaustion: A score of ≥27 indicates a high level of burnout, 19-26 indicates a moderate level of burnout, and <19 indicates a low level of burnout.
- Depersonalization: A score of >10 indicates a high degree of depersonalization, 6-9 indicates a moderate degree of depersonalization, and <6 indicates a low degree of depersonalization.
- Personal accomplishment: A score of 0-30 suggests a low degree of personal accomplishment, 34-39 suggests a moderate degree of personal accomplishment, and >40 suggests a high degree of personal accomplishment.

The UWES is a questionnaire that measures work engagement through 3 aspects: vigor, dedication, and absorption, which are defined by high energy, a sense of significance, and deep involvement in work, respectively. Scores on the UWES are calculated as mean values across the items for each dimension. Based on these mean scores, engagement levels are categorized into 5 distinct groups: very low, low, medium, high, and very high. This categorization allows for a nuanced understanding of work engagement among participants. The UWES shows robust psychometric properties for Spanish medical professionals, with a Cronbach α total reliability score of 0.93 and strong subscale consistencies. It has been proven to be effective for correlating work engagement with health outcomes and job stress, making it a pertinent choice for assessing the positive occupational states in the study [37-40].

### Ethical Considerations

This study was conducted in compliance with the principles outlined in the Declaration of Helsinki (1964) for research involving human subjects, as evaluated and approved by the Badalona Serveis Assistencials’ Research Committee and the Jordi Gol i Gurina Foundation Ethics Committee (approval number: 21/280-P). The committees conducted an ethical review of the project, which included consideration of participant confidentiality, informed consent, and potential risks to participants.

### Statistical Analysis

#### Data Anonymization and Blinding

To protect participant confidentiality while ensuring data integrity, each data set was pseudoanonymized with an identifier code. This measure allowed for an individual response to be tracked without revealing personal information. The principal investigator was the sole individual with access to the decryption key, thus reinforcing the partial blinding of research staff and minimizing potential biases. Statistical analysis was performed with DATAtab (DATAtab e.U.) and SPSS Version 27 (IBM Corp).

#### Normality Tests

The Shapiro-Wilk test was used to determine the distribution of each variable, a necessary step to guide the selection of appropriate statistical tests for the subsequent analyses.

#### Inferential Statistical Analysis and Hypothesis Testing

Following the descriptive summary of the data, inferential statistical analyses were conducted to test the study’s hypothesis that the VR educational program would lead to significant changes in burnout and work engagement scores. The paired *t* test was applied to normally distributed variables to determine if the mean differences in scores before and after the intervention were statistically significant. For data that did not meet the criteria for normal distribution, the Wilcoxon signed-rank test was used to assess median score differences. All tests set the significance threshold at *P*<.05.

## Results

### Demographics

Attrition resulted in a final sample of 83 participants who completed the intervention (5 dropouts from CSSC and 2 from CAP-B; CAP-A retained all its participants). The study achieved a high participation rate of 92% (83/90). Attrition was due to personal circumstances not related to the VR system. Specifically, 1 participant was on maternity leave, 3 were on sick leave, and 3 cited time constraints as the reason for withdrawal. Notably, there were no dropouts attributed to discomfort or adverse reactions to the VR technology.

The demographic characteristics of the participants are summarized in Table 1. The final sample consisted of 83 health care professionals with an average age of 46.39 (range 27-63) years. The cohort included 30 nurses, 15 physicians, and individuals with various other roles such as administrative staff and nursing assistants. Female participants represented the majority of the sample, with 76 female participants compared to 7 male participants. The mean tenure was 17.57 years, with individual tenures ranging from 1 to 42 years, reflecting a wide range of professional experience among the participants. These demographic data offer a comprehensive overview of the study sample, highlighting the diversity in age, professional role, and experience within the health care setting.

**Table 1:** Demographic profile of the study participants.

Professional category	Participants, n	Age (years), mean	Age (years), range	Sex (female/male), n	Tenure (years), mean	Tenure (years), range
Nurses	30	44.93	29-56	27/3	18.90	2-34
Physicians	15	50.80	39-63	12/3	15.33	2-32
Administrative staff	13	48.92	35-59	12/1	16.69	1-36
Nursing assistants	7	45.43	27-58	7/0	17.43	1-29
Occupational therapists	7	46.43	41-57	7/0	24.86	18-42
Social workers	3	41.33	40-43	3/0	7.33	1-16
Psychologists	3	39.33	28-47	3/0	17.67	15-21
Pediatricians	2	36.00	33-39	2/0	7.25	3.5-11
Dentists	2	45.00	41-49	2/0	19.00	13-25
Ward assistants	1	57.00	57	1/0	21.00	21
Overall	83	46.39	27-63	76/7	17.57	1-42

### Descriptive Statistics

Upon establishing the distribution characteristics of the data set, descriptive statistics summarized the data for the 83 participants who completed the VR educational program. The analysis provided an initial overview of the results (Table 2).

**Table 2:** Descriptive statistics of the Maslach Burnout Inventory and Utrecht Work Engagement Scale items among the 83 study participants.

Variable		Mean	Median	SD	Minimum	Maximum
**Emotional exhaustion**						
	Preintervention	22.67	21	10.88	2	51
	Postintervention	17.07	17	9.00	0	42
**Depersonalization**						
	Preintervention	10.04	10	5.07	0	24
	Postintervention	7.37	7	4.34	0	20
**Personal accomplishment**						
	Preintervention	29.72	30	8.64	1	47
	Postintervention	33.08	34	6.70	17	46
**Vigor**						
	Preintervention	22.90	23	5.91	8	35
	Postintervention	24.41	24	5.65	8	36
**Dedication**						
	Preintervention	18.72	19	6.43	6	30
	Postintervention	20.36	22	5.88	8	30
**Absorption**						
	Preintervention	19.86	21	6.58	6	32
	Postintervention	21.71	23	6.39	6	35

### Efficacy of the VR Educational Program

#### Descriptive Analysis

An analysis of MBI and UWES scores after the VR program reflects a potential impact on participants’ professional well-being. Multimedia Appendix 1 presents data indicating a dual effect. There was a reduction in burnout as evidenced by lower emotional exhaustion and depersonalization scores, alongside increased feelings of personal accomplishment. Simultaneously, elevated vigor, dedication, and absorption scores indicated augmented work engagement following the VR intervention.

Following the descriptive analysis, Table 3 provides a detailed breakdown of the distribution of burnout levels among participants both before and after the intervention. The table categorizes participants into low, moderate, and high levels of burnout for each of the following 3 MBI subscales: emotional exhaustion, depersonalization, and personal accomplishment. It presents these categories in both percentage and actual number of participants, offering a clear perspective on the shift in burnout levels after the intervention among the 83 health care professionals involved in the study.

**Table 3:** Distribution of burnout levels among the 83 study participants before and after the intervention.

Variable			High burnout, n (%)		Moderate burnout, n (%)		Low burnout, n (%)
**Emotional exhaustion**							
	Preintervention	27 (33)		24 (29)		32 (39)	
	Postintervention	10 (12)		20 (24)		53 (64)	
**Depersonalization**							
	Preintervention	45 (54)		21 (25)		17 (21)	
	Postintervention	21 (25)		34 (41)		28 (34)	
**Personal accomplishment**							
	Preintervention	44 (53)		26 (31)		13 (16)	
	Postintervention	29 (35)		40 (48)		14 (17)	

#### Statistical Comparisons and Significance

Shapiro-Wilk tests were used to determine the normality of all variables. Emotional exhaustion, depersonalization, personal accomplishment, and vigor, which followed a normal distribution, were analyzed using *t* tests. The nonnormally distributed variables dedication and absorption were analyzed using the Wilcoxon test. Additionally, effect sizes were calculated using Cohen *d* for normally distributed data and Cohen *r* for nonnormally distributed data to quantify the magnitude of observed changes.

#### Correlation and Effect Size Assessments

Correlation assessments were integral to the analysis in order to evaluate relationships between preintervention and postintervention scores. The Pearson correlation was applied to normally distributed data, and the Spearman correlation was used for nonnormally distributed data, with findings reported in Tables 4 and 5. The Cohen *d* and *r* have been provided to reflect the effect sizes and assess the strength of differences, with statistically significant changes (*P*<.05) in all variables.

**Table 4:** Summary of preintervention and postintervention analysis results for normally distributed variables.

Variable	Preintervention result, mean (SD)	Postintervention result, mean (SD)	*t* test		Effect size, Cohen *d*	Pearson *r*	Pearson *P*
			*t* (*df*)	*P* value (2-tailed)			
Emotional exhaustion	22.67 (10.88)	17.07 (9.00)	5.58 (82)	<.001	0.61	0.59	<.001
Depersonalization	10.04 (5.07)	7.37 (4.34)	4.67 (82)	<.001	0.51	0.40	<.001
Personal accomplishment	29.72 (8.64)	33.08 (6.70)	−3.62 (82)	<.001	0.40	0.41	<.001
Vigor	22.65 (5.98)	24.66 (5.50)	−3.77 (82)	<.001	0.41	0.64	<.001

**Table 5:** Summary of preintervention and postintervention analysis results for nonnormally distributed variables.

Variable	Preintervention result, median (IQR)	Postintervention result, median (IQR)	Wilcoxon test				Effect size, Cohen *r*		Spearman *r*		Spearman *P*
			*W*	*z*	*P* value (2-tailed)						
Dedication	19 (12)	22 (8)	580	−3.63	<.001	0.41		0.73		<.001	
Absorption	20 (10)	23 (9.5)	785.5	−3.52	<.001	0.40		0.67		<.001	

### Epidemiologic Factors

Spearman correlation analysis was employed to explore relationships between age, tenure, and the main study variables, and it revealed no significant correlations. Similarly, point biserial correlation analysis indicated no significant correlations between sex and the study variables both before and after the intervention.

The influence of professional categories on questionnaire outcomes was assessed using η² values (Table 6). The η² values ranged from 0.01 to 0.24 and indicated the proportion of variance in each measure that can be attributed to the professional category. Higher η² values, such as those for preintervention depersonalization, suggested a more significant variance related to the professional category, while other measures exhibited a lower degree of variance due to professional categorization, reflecting a range of impacts across different professional categories.

The ANOVA analysis provided deeper insights into the intervention’s impact, examining changes in the MBI and UWES scores. This analysis encompassed overall changes and variations across different professional categories. Detailed statistical outcomes, including sums of squares, mean squares, *F* values, and *P* values, are presented in Table 7, highlighting the statistical significance of the observed changes.

**Table 6:** Maslach Burnout Inventory and Utrecht Work Engagement Scale data across different professional categories.

Professional category	Emotional exhaustion, mean (SD)			Depersonalization, mean (SD)			Personal accomplishment, mean (SD)			Vigor, mean (SD)			Dedication, mean (SD)			Absorption, mean (SD)	
	Pre^a^	Post^b^	Pre		Post	Pre		Post	Pre		Post	Pre		Post	Pre		Post
Nurses	20.37 (8.78)	18.77 (8.22)	9.97 (4.43)		8.00 (4.10)	30.03 (8.26)		33.73 (7.23)	21.90 (6.83)		23.37 (5.84)	18.57 (6.78)		20.23 (5.66)	19.40 (7.37)		21.37 (6.35)
Physicians	26.13 (12.70)	19.93 (9.90)	10.13 (4.66)		7.80 (4.72)	29.40 (5.59)		31.53 (7.62)	21.60 (5.33)		24.60 (5.26)	18.33 (6.20)		21.60 (5.53)	20.80 (6.11)		24.07 (4.38)
Administrative staff	22.00 (7.97)	15.77 (9.36)	13.15 (3.93)		8.00 (4.81)	27.23 (8.63)		31.23 (5.95)	24.00 (4.93)		27.08 (4.96)	16.38 (6.46)		18.69 (6.13)	19.77 (6.31)		22.31 (7.09)
Nursing assistants	22.43 (10.50)	16.00 (12.49)	11.00 (4.90)		7.71 (4.54)	25.29 (14.28)		32.00 (8.70)	23.29 (6.42)		26.14 (6.15)	20.43 (7.28)		21.43 (6.85)	19.00 (7.79)		22.14 (7.65)
Occupational therapists	17.86 (12.42)	10.57 (4.08)	9.14 (7.36)		4.57 (2.23)	33.43 (6.75)		35.43 (2.64)	25.00 (6.08)		24.57 (4.72)	20.43 (7.74)		21.29 (4.75)	19.00 (6.93)		19.57 (7.59)
Psychologists	36.33 (4.16)	14.33 (6.03)	3.00 (3.61)		3.33 (1.53)	27.67 (10.21)		35.67 (5.03)	22.33 (2.89)		26.67 (1.53)	21.67 (2.52)		22.00 (1.00)	21.33 (9.81)		23.33 (6.35)
Social workers	15.33 (8.08)	11.67 (6.81)	2.67 (2.52)		4.33 (4.51)	35.67 (12.70)		33.33 (8.14)	19.67 (7.57)		23.00 (5.57)	18.67 (10.21)		20.33 (8.08)	20.33 (8.14)		22.33 (6.43)
Pediatricians	43.50 (10.61)	27.00 (5.66)	13.50 (2.12)		11.50 (7.78)	29.00 (5.66)		33.00 (4.24)	23.50 (6.36)		17.00 (4.24)	16.00 (5.66)		12.50 (4.95)	18.00 (5.66)		17.00 (2.83)
Ward assistants	26.50 (20.51)	12.50 (6.36)	10.50 (6.36)		5.50 (3.54)	38.00 (11.31)		38.00 (4.24)	25.00 (9.90)		28.00 (4.24)	23.00 (7.07)		21.50 (4.95)	18.50 (7.78)		21.00 (7.07)
Dentists	16.00 (N/A^c^)	7.00 (N/A)	5.00 (N/A)		8.00 (N/A)	36.00 (N/A)		34.00 (N/A)	26.00 (N/A)		31.00 (N/A)	19.00 (N/A)		27.00 (N/A)	17.00 (N/A)		21.00 (N/A)
η^d^	0.47	0.39	0.49		0.35	0.32		0.24	0.23		0.37	0.23		0.31	0.12		0.24
η^2d^	0.22	0.15	0.24		0.13	0.1		0.06	0.05		0.14	0.05		0.09	0.01		0.06

^a^Pre: preintervention.

^b^Post: postintervention.

^c^N/A: not applicable.

^d^η and η² values indicate the variance due to professional categorization.

**Table 7:** ANOVA results for preintervention and postintervention measures and professional categories.

Variable			Sum of squares		Degrees of freedom (*df*)		Mean squares		*F*		*P* value
**Emotional exhaustion**											
	Preintervention/postintervention	1302.56		1		1302.56		37.07		<.001	
	Professional category	2294.19		9		254.91		1.75		.09	
	A×B	861.83		9		95.76		2.73		.008	
**Depersonalization**											
	Preintervention/postintervention	294.22		1		294.22		21.89		<.001	
	Professional category	567.48		9		63.05		2.33		.02	
	A×B	126.04		9		14.00		1.04		.42	
**Personal accomplishment**											
	Preintervention/postintervention	468.92		1		468.92		12.37		.001	
	Professional category	656.22		9		72.91		0.86		.57	
	A×B	168.51		9		18.72		0.49		.87	
**Vigor**											
	Preintervention/postintervention	168.01		1		168.01		14.59		<.001	
	Professional category	360.04		9		40.00		0.72		.69	
	A×B	131.10		9		14.57		1.27		.27	
**Dedication**											
	Preintervention/postintervention	128.41		1		128.41		13.11		.001	
	Professional category	358.90		9		39.88		0.58		.81	
	A×B	84.81		9		9.42		0.96		.48	
**Absorption**											
	Preintervention/postintervention	208.41		1		208.41		12.78		.001	
	Professional category	194.98		9		21.66		0.29		.98	
	A×B	34.49		9		3.83		0.24		.99	

## Discussion

### Principal Findings

In this pilot study, we implemented an 8-week VR educational program focused on mindfulness and emotional management for health care professionals. The results suggest that the program can positively affect burnout symptoms and work engagement in this group. This inference is drawn from the significant reductions observed in burnout symptoms and enhancements in work engagement metrics after the intervention (Tables 2-5 and Multimedia Appendix 1). Complementing the observed reductions in burnout symptoms, the VR educational program also appears to have positively influenced work engagement (Tables 4 and 5). These shifts are especially prominent in the dimensions of dedication and absorption, highlighting the VR program’s potential in enhancing aspects of work engagement that relate to a sense of significance and deep involvement in work.

The reduction in score variability after the intervention, as shown in Tables 4 and 5, implies a standardized effectiveness of the VR program in mitigating burnout symptoms. This uniform decrease in scores like emotional exhaustion and depersonalization scores highlights the VR program’s consistent impact across participants. The effect sizes, as analyzed from Tables 4 and 5, reveal a more significant impact of the VR intervention in reducing negative burnout aspects, particularly emotional exhaustion and depersonalization, than in enhancing positive work attributes.

### Differential Impact Across Professional Categories

The VR program’s effects varied among health care roles, as seen in the η² values in Table 6. High variability in emotional exhaustion and depersonalization suggests that roles like psychologists and pediatricians might be more vulnerable to certain burnout aspects. Conversely, measures like vigor and absorption showed more uniform responses across roles. The ANOVA analysis revealed significant variation for emotional exhaustion across categories, emphasizing the need for tailored interventions in health care (Table 7). However, the findings should be approached cautiously due to the limited sample size in some categories, needing further research for conclusive results.

### Comparison With Prior Work

The improvement in positive occupational states is a key outcome, given the critical role of engagement in the overall well-being and job performance of health care professionals [41]. The successful application of VR in mindfulness and emotional management training is consistent with existing literature [12,13], underscoring its potential as a vital psychosocial intervention tool in health care settings. This aligns with the findings of Lee and Cha [42], indicating the need for refined VR strategies to balance reducing burnout and boosting work engagement in health care.

### Strengths and Implications

This pilot study has significant strengths and yields insights with practical implications for addressing burnout in health care professionals. The low dropout rate reinforces user engagement and the potential of VR as a sustainable educational tool, as observed in other research efforts [14-16,43]. The adherence rate of 92% (83/90) along with minimal reports of discomfort from VR use demonstrates its viability in a clinical setting. These factors are encouraging, especially considering the need for ergonomic consideration highlighted by the ease of resolving discomfort with simple adjustments.

The study’s VR intervention was designed with a focus on user comfort, drawing from the mitigation strategies outlined in the VR Considerations subsection of the Methods section. This careful design likely contributed to the positive reception and ease of use reported by participants, underscoring the potential of VR in supporting World Health Organization–endorsed strategies for stress management in clinical environments [8].

### Limitations

The promising results obtained in this pilot study must be viewed within the context of some methodological constraints. First, the lack of a control group in the study design precludes a definitive conclusion regarding the causality of observed changes, suggesting that further studies with control groups are necessary to corroborate the VR program’s effectiveness. Second, the reliance on convenience sampling poses a risk for selection bias, and future studies may benefit from randomized sampling to ensure broader applicability of the results. Third, the study’s participant demographic, with a significant underrepresentation of male participants, reflects the female majority typical in health care settings, particularly in our primary care centers where approximately 79.6% of our health care workers are female according to data provided by our Human Resources department in November 2023. This gender imbalance limits the generalizability of our findings across all genders. Future research should aim to include a more balanced gender distribution when possible, exploring if VR educational programs have differential effects on various demographic groups. Fourth, the sample size, which is appropriate for a preliminary exploration into VR-based interventions, is nonetheless insufficient for establishing definitive efficacy, underscoring the need for larger more representative studies. Resource-related phased implementation may have introduced variability in delivery, which was mitigated in later stages but could have affected initial participant experience. Future studies should increase the sample size to provide more robust evidence of efficacy. It is also recommended to standardize the implementation process across all phases to minimize variability and improve the reliability of the results.

### Conclusions

This pilot study provides initial indications that an 8-week VR-based educational program may positively influence burnout and work engagement among health care professionals. Our findings suggest a reduction in burnout symptoms, as measured by the MBI, and an increase in work engagement, as indicated by the UWES. While the data hint at VR’s promise for reducing burnout symptoms and fostering work engagement, the results should be viewed as preliminary. The interpretive value of the findings is limited by the sample size and the absence of a control group, despite the application of rigorous statistical analyses to assess the intervention’s impact.

To enhance future assessments and maintain equitable access to potential benefits, subsequent research could adopt a crossover study design. Such a design would involve randomizing participants into initial control and intervention groups, with a subsequent exchange of roles following a predetermined washout period. This methodological approach would allow for a comprehensive evaluation of the VR program’s impact by ensuring that each participant acts as their own control, thereby reinforcing the strength of the evidence gathered while ensuring access to the intervention.

This study contributes to the literature on the use of VR technology in education and health care, highlighting the potential of digital health interventions in disease prevention and health promotion, and emphasizing the importance of prioritizing the well-being of health care professionals as a main requirement for the survival of the health care system.

## Appendix

Multimedia Appendix 1 Mean scores of the Maslach Burnout Inventory and Utrecht Work Engagement Scale.

## Abbreviations

MBI: Maslach Burnout Inventory
UWES: Utrecht Work Engagement Scale
VR: virtual reality

## Data Availability

The data sets generated or analyzed during this study are available from the corresponding author upon reasonable request.

## References

[1] Wright T, Mughal F, Babatunde O, Dikomitis L, Mallen C, Helliwell T (2022). Burnout among primary health-care professionals in low- and middle-income countries: systematic review and meta-analysis. Bull World Health Organ.

[2] Hodkinson A, Zhou A, Johnson J, Geraghty K, Riley R, Zhou A, Panagopoulou E, Chew-Graham CA, Peters D, Esmail A, Panagioti M (2022). Associations of physician burnout with career engagement and quality of patient care: systematic review and meta-analysis. BMJ.

[3] García-Iglesias JJ, Gómez-Salgado J, Fagundo-Rivera J, Romero-Martín M, Ortega-Moreno M, Navarro-Abal Y (2022). Factores predictores de los niveles de burnout y work engagement en médicos y enfermeras: una revisión sistemática. Revista Española Salud Pública.

[4] Stodolska A, Wójcik G, Barańska I, Kijowska V, Szczerbińska K (2023). Prevalence of burnout among healthcare professionals during the COVID-19 pandemic and associated factors - a scoping review. Int J Occup Med Environ Health.

[5] Seda-Gombau G, Montero-Alía J, Moreno-Gabriel E, Torán-Monserrat P (2021). Impact of the COVID-19 Pandemic on Burnout in Primary Care Physicians in Catalonia. Int J Environ Res Public Health.

[6] Han S, Shanafelt T, Sinsky C, Awad K, Dyrbye L, Fiscus L, Trockel M, Goh J (2019). Estimating the attributable cost of physician burnout in the United States. Ann Intern Med.

[7] Sinsky CA, Shanafelt TD, Dyrbye LN, Sabety AH, Carlasare LE, West CP (2022). Health care expenditures attributable to primary care physician overall and burnout-related turnover: a cross-sectional analysis. Mayo Clin Proc.

[8] (2022). Guidelines on mental health at work. World Health Organization.

[9] Lomas T, Medina J, Ivtzan I, Rupprecht S, Eiroa-Orosa F (2018). A systematic review of the impact of mindfulness on the well-being of healthcare professionals. J Clin Psychol.

[10] Maresca G, Corallo F, Catanese G, Formica C, Lo Buono V (2022). Coping strategies of healthcare professionals with burnout syndrome: a systematic review. Medicina (Kaunas).

[11] Kabat-Zinn J (1994). Wherever You Go, There You Are: Mindfulness Meditation in Everyday Life.

[12] Selič-Zupančič P, Klemenc-Ketiš Z, Onuk Tement S (2023). The Impact of Psychological Interventions with Elements of Mindfulness on Burnout and Well-Being in Healthcare Professionals: A Systematic Review. J Multidiscip Healthc.

[13] Cepeda-Lopez A, Solís Domínguez L, Villarreal Zambrano S, Garza-Rodriguez I, Del Valle A, Quiroga-Garza A (2023). A comparative study of well-being, resilience, mindfulness, negative emotions, stress, and burnout among nurses after an online mind-body based intervention during the first COVID-19 pandemic crisis. Front Psychol.

[14] Navarro-Haro M, López-Del-Hoyo Y, Campos D, Linehan M, Hoffman H, García-Palacios A, Modrego-Alarcón M, Borao L, García-Campayo J (2017). Meditation experts try Virtual Reality Mindfulness: A pilot study evaluation of the feasibility and acceptability of Virtual Reality to facilitate mindfulness practice in people attending a Mindfulness conference. PLoS One.

[15] Chandrasiri A, Collett J, Fassbender E, De Foe A (2019). A virtual reality approach to mindfulness skills training. Virtual Reality.

[16] Ma J, Zhao D, Xu N, Yang J (2023). The effectiveness of immersive virtual reality (VR) based mindfulness training on improvement mental-health in adults: A narrative systematic review. Explore (NY).

[17] Yang H, Cai M, Diao Y, Liu R, Liu L, Xiang Q (2022). How does interactive virtual reality enhance learning outcomes emotional experiences? A structural equation modeling approach. Front Psychol.

[18] Moro C, Štromberga Z, Raikos A, Stirling A (2017). The effectiveness of virtual and augmented reality in health sciences and medical anatomy. Anat Sci Educ.

[19] Asad MM, Naz A, Churi P, Tahanzadeh MM (2021). Virtual Reality as Pedagogical Tool to Enhance Experiential Learning: A Systematic Literature Review. Education Research International.

[20] Barteit S, Lanfermann L, Bärnighausen Till, Neuhann F, Beiersmann C (2021). Augmented, mixed, and virtual reality-based head-mounted devices for medical education: systematic review. JMIR Serious Games.

[21] Pascual K, Fredman A, Naum A, Patil C, Sikka N (2023). Should mindfulness for health care workers go virtual? A mindfulness-based intervention using virtual reality and heart rate variability in the emergency department. Workplace Health Saf.

[22] Meese MM, O'Hagan EC, Chang TP (2021). Healthcare provider stress and virtual reality simulation: a scoping review. Simul Healthc.

[23] Lindner P, Miloff A, Hamilton W, Carlbring P (2019). The potential of consumer-targeted virtual reality relaxation applications: descriptive usage, uptake and application performance statistics for a first-generation application. Front Psychol.

[24] Oing T, Prescott J (2018). Implementations of virtual reality for anxiety-related disorders: systematic review. JMIR Serious Games.

[25] Navarro-Haro M, Modrego-Alarcón Marta, Hoffman H, López-Montoyo Alba, Navarro-Gil M, Montero-Marin J, García-Palacios Azucena, Borao L, García-Campayo Javier (2019). Evaluation of a mindfulness-based intervention with and without virtual reality dialectical behavior therapy mindfulness skills training for the treatment of generalized anxiety disorder in primary care: a pilot study. Front Psychol.

[26] Lu F, Ratnapalan S (2023). Burnout interventions for resident physicians: a scoping review of their content, format, and effectiveness. Arch Pathol Lab Med.

[27] Souchet AD, Lourdeaux D, Burkhardt J, Hancock PA (2023). Design guidelines for limiting and eliminating virtual reality-induced symptoms and effects at work: a comprehensive, factor-oriented review. Front Psychol.

[28] Weech S, Kenny S, Barnett-Cowan M (2019). Presence and Cybersickness in Virtual Reality Are Negatively Related: A Review. Front Psychol.

[29] Yao R, Heath T, Davies A, Forsyth T, Mitchell N, Hoberman P (2014). Oculus VR best practices guide. Oculus VR.

[30] Gavgani A, Nesbitt K, Blackmore K, Nalivaiko E (2017). Profiling subjective symptoms and autonomic changes associated with cybersickness. Auton Neurosci.

[31] Maslach C, Schaufeli W, Leiter M (2001). Job burnout. Annu Rev Psychol.

[32] Edú-Valsania Sergio, Laguía Ana, Moriano JA (2022). Burnout: a review of theory and measurement. Int J Environ Res Public Health.

[33] Forné Carles, Yuguero O (2022). Factor structure of the Maslach Burnout Inventory Human Services Survey in Spanish urgency healthcare personnel: a cross-sectional study. BMC Med Educ.

[34] Cañadas-de la Fuente GA, San Luis C, Lozano LM, Vargas C, García I, de la Fuente EI (2014). Evidencia de validez factorial del Maslach Burnout Inventory y estudio de los niveles de burnout en profesionales sanitarios. Revista Latinoamericana de Psicología.

[35] Hernández Vargas CI, Llorens Gumbau S, Rodríguez Sánchez AM Burnout en personal sanitario: validación de la escala MBI en México. Forum de recerca.

[36] Maslach C, Jackson SE, Leiter MP, Zalaquett CP, Wood RJ (1997). Maslach Burnout Inventory. Evaluating stress: A book of resources.

[37] Utrecht Work Engagement Scale. Occupational Health Psychology Unit, Utrecht University.

[38] Torabinia M, Mahmoudi S, Dolatshahi M, Abyaz MR (2017). Measuring engagement in nurses: the psychometric properties of the Persian version of Utrecht Work Engagement Scale. Med J Islam Repub Iran.

[39] Domínguez-Salas S, Rodríguez-Domínguez C, Arcos-Romero A, Allande-Cussó R, García-Iglesias J, Gómez-Salgado J (2022). Psychometric Properties of the Utrecht Work Engagement Scale (UWES-9) in a Sample of Active Health Care Professionals in Spain. Psychol Res Behav Manag.

[40] Ruiz-Frutos C, Ortega-Moreno M, Soriano-Tarín G, Romero-Martín M, Allande-Cussó R, Cabanillas-Moruno JL, Gómez-Salgado J (2021). Psychological Distress Among Occupational Health Professionals During Coronavirus Disease 2019 Pandemic in Spain: Description and Effect of Work Engagement and Work Environment. Front Psychol.

[41] Han S (2023). Nurses' job crafting, work engagement, and well-being: a path analysis. BMC Nurs.

[42] Lee M, Cha C (2023). Interventions to reduce burnout among clinical nurses: systematic review and meta-analysis. Sci Rep.

[43] Slater M, Sanchez-Vives Mv (2016). Enhancing our lives with immersive virtual reality. Front Robot AI.

