# Supplementary material for: Immediate Impact of an 8-Week Virtual Reality Educational Program on Burnout and Work Engagement Among Health Care Professionals: Pre-Post Pilot Study
Source: JMIR XR Spat Comput. 2024 Apr 25;1:e55678. doi: 10.2196/55678 (PMC12671320; doi:10.2196/55678)
Supplement: Multimedia Appendix 1 [file xr_v1i1e55678_app1.pdf]

Multimedia Appendix 1. Mean scores of the Maslach Burnout Inventory and Utrecht Work Engagement Scale.

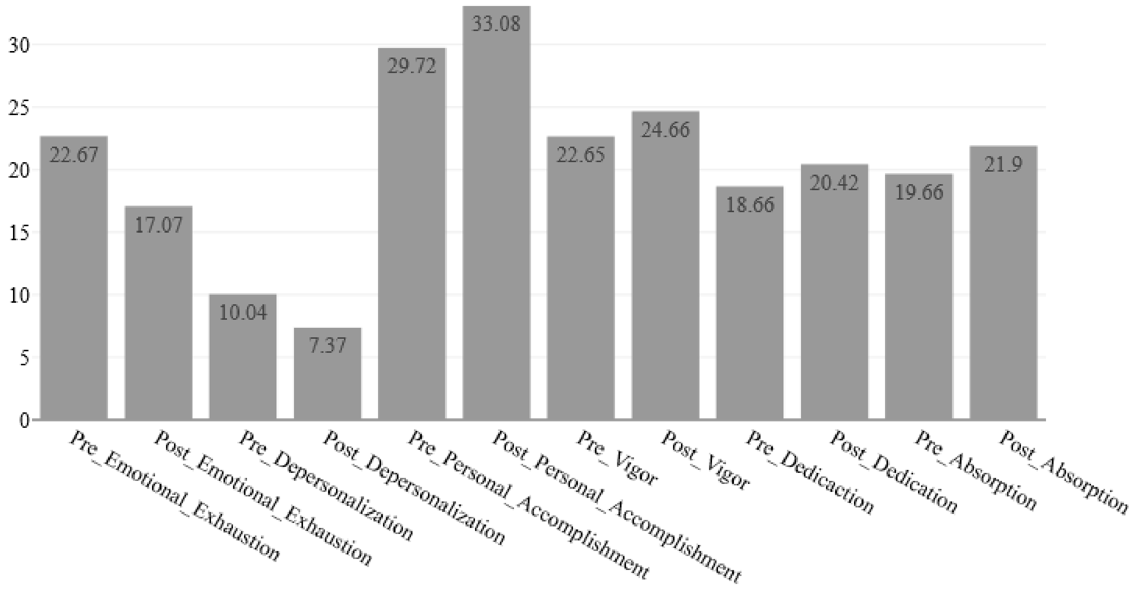

This figure illustrates the average scores across the three domains of both scales - Emotional Exhaustion, Depersonalization, and Personal Accomplishment from the MBI, and Vigor, Dedication, and Absorption from the UWES - before and after the 8-week intervention. Post-intervention, there is a noticeable decrease in scores for Emotional Exhaustion and Depersonalization, and an increase in Personal Accomplishment, indicating a significant reduction in burnout levels. Simultaneously, scores in Vigor, Dedication, and Absorption increased, suggesting enhanced work engagement among participants.
